# Physical activity intervention improves executive function in children with autism spectrum disorder: a meta-analysis

**DOI:** 10.3389/fped.2026.1693801

**Published:** 2026-03-12

**Authors:** Yongjing Li, Chungang Gao, Shuhua Song

**Affiliations:** School of Physical Education, Yunnan Normal University, Kunming, Yunnan, China

**Keywords:** autism spectrum disorder, executive function, physical activity, inhibition, working memory, cognitive flexibility, meta-analysis

## Abstract

**Objective:**

This systematic review and meta-analysis evaluated the effects of various physical activities on executive function (EF) and its components—inhibition, working memory, and cognitive flexibility—in children with ASD.

**Methods:**

Six databases (Cochrane Library, PubMed, Embase, Web of Science, ScienceDirect, CBMdisc) were searched from inception to December 2025. Additional studies were identified through reference list screening. We used RevMan 5.4 and the GRADE framework to assess risk of bias and quality grades, and Stata 18 to generate forest and funnel plots for data analysis.

**Results:**

Fourteen RCTs met the inclusion criteria. Physical activity resulted in at least moderate improvements in EF and all subdomains. Notably, FMS training administered four times per week for 18 weeks produced a large effect on EF [SMD = 2.62, 95% CI = 1.50–3.74]. In comparison, digital motion-sensing games showed the largest effects on inhibition [SMD = 1.38, 95% CI = 0.52–2.25], working memory [SMD = 0.89, 95% CI = 0.08–1.70], and cognitive flexibility [SMD = 0.88, 95% CI = 0.36–1.39]. A protocol of 15 min per session, two to three sessions per week for 18 weeks, achieved at least moderate improvements in inhibition and cognitive flexibility, while working memory showed a large effect by eight weeks.

**Conclusions:**

FMS training conducted four times per week for 18 weeks provides the greatest overall benefit. Fifteen-minute digital motion-sensing game sessions, two to three times per week for 18 weeks, are most effective for improving inhibition and cognitive flexibility, and working-memory improvements are observable within four weeks. Further research with larger sample sizes is recommended.

**Systematic Review Registration:**

https://www.crd.york.ac.uk/PROSPERO/view/CRD420251130511, PROSPERO CRD420251130511.

## 1 Introduction

Autism Spectrum Disorder, also known as ASD, is a term used to describe a specific combination of impairments in social communication skills, repetitive behaviors, restricted interests, and/or sensory behaviors. These characteristics typically begin to manifest in early childhood (1). In adulthood, these characteristics become more pronounced compared to neurotypical adults, with significantly increased levels of anxiety and loneliness, and a decline in quality of life. Research has confirmed that the prevalence of ASD is approximately 1% among school-aged children, with reporting rates continuing to rise (2, 3). Consequently, numerous theoretical mechanisms have been proposed to explain ASD symptoms, among which the executive dysfunction theory has been supported by substantial evidence demonstrating a significant association between ASD related behavioral manifestations and impaired executive processes. This theory holds that the behavioral and movement disorders in children with ASD are caused by dysfunction of a system comprising bilateral nerve structures, including the central limbic cortical loop located in the prefrontal and medial temporal lobes, the neostriatum, and the anteromedial nuclear group of the thalamus (4).

Executive functions (EF) are higher-order cognitive abilities that serve as essential higher-level control processes for guiding behavior in dynamic environments and are associated with frontal lobe activity. It comprises three core components: inhibition, working memory, and cognitive flexibility, which are closely linked to clinical features in children with ASD, such as social symptoms, repetitive and stereotyped behaviors, persistence, and compulsive behaviors (5, 6). Specifically, restricted repetitive behaviors can be attributed to deficits in cognitive flexibility. In contrast, deficits in initiation, working memory, planning, organization, and monitoring are significantly associated with social functioning in children with ASD (7, 8). EF can be improved through practice, and children with EF deficits benefit more from EF interventions than typically developing children. Therefore, early intervention targeting EF may help minimize the differences between children with ASD and typically developing children.

Physical activity, defined as regular engagement in sports or exercise aimed at improving or maintaining physical health, has been repeatedly shown in previous studies to have a significant impact on children's EF (9, 10). To date, ongoing research has further explored the relationship between various types of physical activity and EF, as well as the underlying mechanisms, such as the sensitivity of EF to chronic aerobic exercise, which is associated with the inherent cognitive demands of physical activity (11). Another experimental design focused on acute exercise, using different types of physical activity interventions to demonstrate that complex movements rely on prefrontal cortex-related EFs, thereby enhancing prefrontal neural function (12). Furthermore, the physiological responses to exercise and the subsequent cellular and molecular changes directly benefit the brain (13). Therefore, recent meta-analyses have aimed to synthesize multiple studies to specifically analyze the benefits of exercise interventions on EF in children with ASD (14, 15). However, existing studies have not comprehensively analyzed the specific types and intensities of interventions. As research findings continue to evolve, new intervention measures and evidence-based practices have emerged. Consequently, this study aims to conduct a comprehensive analysis of existing research on physical activity interventions for EF in children with ASD to identify the most effective intervention strategies.

## 2 Data and methods

### 2.1 Study registration

This study was registered on the PROSPERO platform, with the registration number: CRD420251130511.

### 2.2 Literature search

#### 2.2.1 Searchers and search period

The first and second authors conducted independent searches in accordance with a predefined protocol. The search period covered all databases from their inception to December 2025.

#### 2.2.2 Search databases

The Cochrane Library, PubMed, Embase, Web of Science, ScienceDirect, CBMdisc, and Google Scholar. No targeted search of grey literature (e.g., dissertations, conference abstracts, unpublished trial data, government reports) was conducted in this study.

#### 2.2.3 Search terms and search strategy

(1) Intervention Target: “Autism Spectrum Disorder*”, “Autistic Spectrum Disorder*”, “Disorder, Autistic Spectrum”, “Autism”, “Executive Function”, “Working Memory”, “Inhibitory Control”, “Cognitive Flexibility”, “Executive control”, “Inhibition Control”, “Cognition”, “Cognitive”; (2) Intervention measures: “physical activity”, “exercise”, “sports games”, “basketball”, “soccer”, “martial arts”, “Movement”, “swimming”, ‘dance’, “Dance”. Using PubMed as an example, the search strategy is illustrated in Supplementary Figure S1.

### 2.3 Inclusion and exclusion criteria

#### 2.3.1 Inclusion criteria

(1) Study Population: Children aged 0–18 years diagnosed with ASD according to the Diagnostic and Statistical Manual of Mental Disorders, 5th Edition (DSM−5) (16); (2) Intervention: Including various forms of physical activities; (3) Between physical activity groups or between a physical activity group and a no-intervention group; (4) Outcome Measures: Assessment of overall EF score, inhibition, and working memory before and after the intervention; (5) Study Design: Included studies were randomized trials.

#### 2.3.2 Exclusion criteria

(1) Study subjects with other diseases; (2) Review articles or conference papers; (3) Full text unavailable; (4) Studies lacking extractable pre- and pos-intervention data in the form of mean ± SD (including articles reporting only median, interquartile range, standard error, or Z-score, or where back-calculation of mean ± SD is impossible); (5) Publications not in English or Chinese.

### 2.4 Literature screening and data extraction

Two researchers screened the literature according to the pre-formulated inclusion criteria. In case of disagreement, the two discussed it. If it still couldn't be resolved, they consulted the corresponding author to determine whether the literature met the inclusion criteria. First, the titles and abstracts of the articles were reviewed for preliminary screening. Subsequently, the full text was read to determine which studies met the inclusion criteria. The extraction of data included the first author of the included study, publication year, country, demographic data, total sample size, outcome indicators, intervention measures, intervention protocols (frequency, dose, cycle), and methodological quality information.

### 2.5 Quality assessment

This study used the Cochrane Risk of Bias Assessment Tool for evaluation. The tool assesses risk of bias across six domains: selection bias (random sequence generation, allocation concealment), performance bias (blinding of participants and personnel), measurement bias (blinding of outcome assessment), follow-up bias (incomplete outcome data), reporting bias (selective outcome reporting), and other biases. Each indicator was rated as “low risk of bias,” “unclear,” or “high risk of bias”.

### 2.6 Statistical analysis

First, outcome measures examined EF, inhibition, working memory, and cognitive flexibility in individuals with ASD, treating all data as continuous variables. Effect sizes were calculated as Standardized Mean Differences (SMDs) with 95% confidence intervals. If outcome measures were reverse-scored (see Supplementary Table S1) and caused inconsistent effect directions, we multiplied their SMDs by −1 to maintain consistency. This approach aligned all SMDs for directional uniformity across the meta-analysis. We assessed risk of bias using RevMan 5.4 and rated evidence quality with the GRADE system. Forest plots in Stata 18 illustrated results from individual studies and the pooled effect size. Subgroup analyses explored differences in efficacy and potential sources of heterogeneity. Finally, we generated a funnel plot using the Stata metabias command and checked for publication bias with Egger's test. If bias was present (*P* < 0.05), we applied nonparametric trim-and-fill analysis to address heterogeneity and support our findings.

## 3 Results

### 3.1 Literature search and process

**Figure 1:**
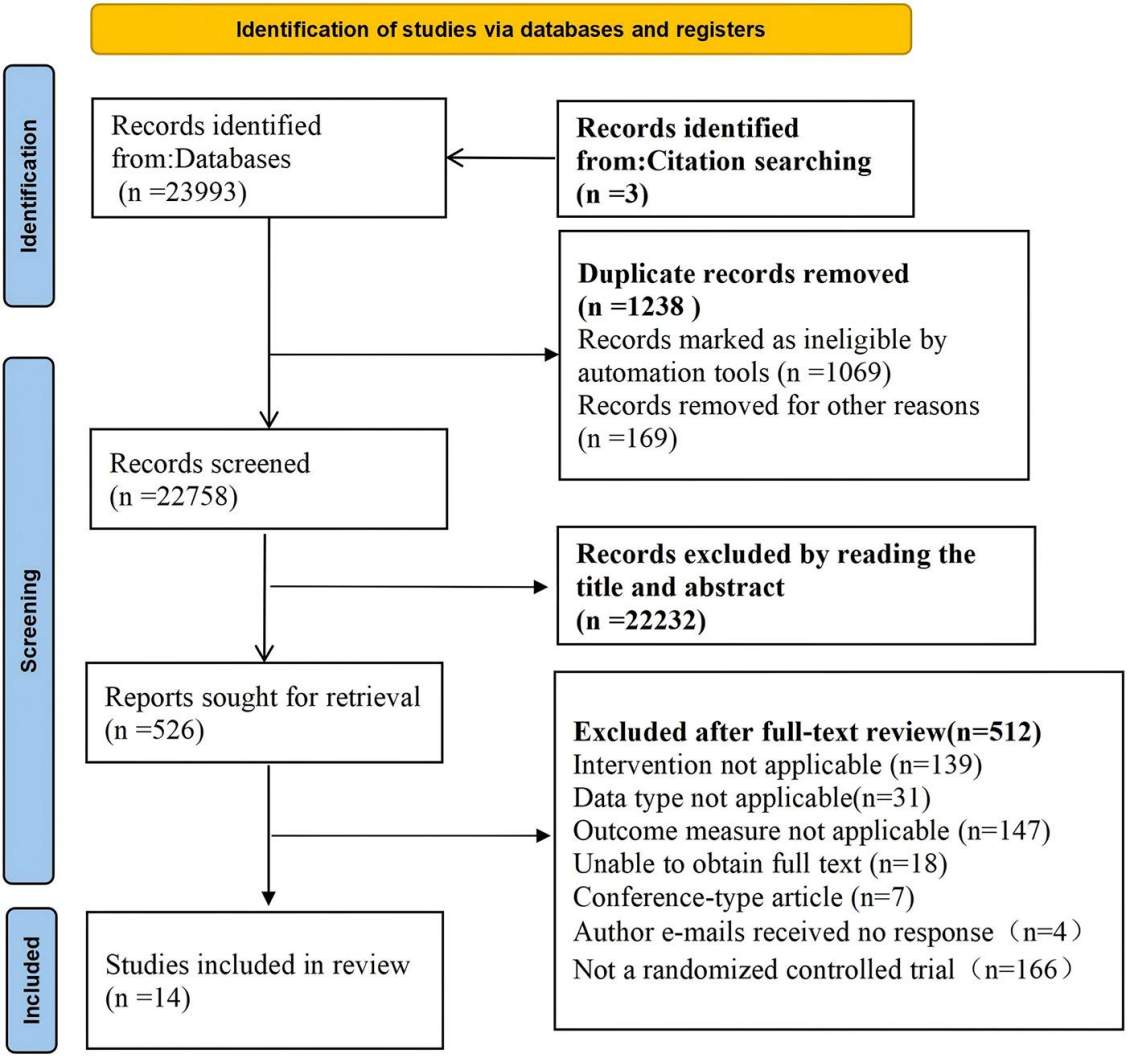
Literature screening flowchart.

This study ultimately included 14 randomized trials. The detailed literature screening process is shown in Figure 1.

### 3.2 Basic characteristics of the included studies

The basic characteristics of the 14 included studies are presented in Table 1. These studies were conducted in China (*n* = 7), the United States (*n* = 1), Italy (*n* = 2), Iran (*n* = 3), and South Korea (*n* = 1), with total sample sizes ranging from 16 to 60 participants. The average age of the intervention subjects in the experimental and control groups was 7.26 ± 1.54 and 8.20 ± 1.38, respectively, with the highest age being 14.42 ± 5.14 and 14.17 ± 5.09, respectively; males predominated. The intervention lasted 4 to 18 weeks, with 2–5 sessions per week, and each session lasted 15–70 min.

**Table 1:** Characteristics of included studies.

Study	Country	Mean age (T/C)	Sample size (T/C)	M/W (T/C)	Intervention measures (T/C)		Intervention dose	Outcome indicator
Tse (17) 2019	China	10.11 ± 1.2/9.81 ± 1.17	40 (19/21)	14/5 18/3	Basketball skill acquisition	No intervention	45 min/s, 2 s/wk, 12wk	GNG, BDS
Greco (21) 2020	Italy	9.25 ± 0.97/ 9.42 ± 0.90	24 (12/12)	10/2 10/2	Gamified exercise	No intervention	70 min/s, 2 s/wk, 12wk	BRIEF
Greco2 (22) 2020	Italy	9.07 ± 1.00/ 9.43 ± 1.02	28 (14/14)	12/2 12/2	Karate training	No intervention	45 min/s, 2 s/wk, 12wk	BRIEF
Wang (19) 2020	China	5.11 ± 0.65/ 4.70 ± 0.70	33 (18/15)	15/3 13/2	Basketball skill acquisition	No intervention	40 min/s, 5 s/wk, 12wk	BRIEF
Milajerdi (23) 2021	Iran	7.95 ± 1.60 8.15 ± 1.50 8.45 ± 1.43	60 (20/20/20)	19/1 19/1 19/1	T1: Gamified exercise T2: digital motion-sensing game	No intervention	35 min/s, 3 s/wk, 8wk	WCST
Chen (24) 2024	China	5.27 ± 0.70/5.07 ± 1.10	30 (15/15)	12/3 13/2	Gamified exercise	No intervention	30 min/s, 6 s/wk, 8wk	Day/Night Task
Nekar (30) 2022	South Korea	14.42 ± 5.14 14.17 ± 5.09	24 (12/12)	10/2 12/0	Digital motion-sensing game	Cognitive training	15 min/s, 2s/wk, 4wk	GNG, DSF, Stroop Test
Chan (18) 2013	China	11.28 ± 3.9/ 12.42 ± 3.25	40 (20/20)	19/1 17/3	Nei Yang Gong	Progressive Muscle Relaxation	60 min/s, 2 s/wk, 4wk	GNG
Faraji (27) 2023	Iran	7.26 ± 1.54/ 8.20 ± 1.38	40 (20/20)	-	Aquatic exercise	No intervention	45 min/s, 3 s/wk, 8wk	WCST
Pan (25) 2016	China	9.68 ± 1.61/ 8.49 ± 1.76	22 (11/11)	-	Table tennis skill acquisition	No intervention	70 min/s, 2 s/wk, 12wk	WCST
Alooche (28) 2025	Iran	9.46 ± 1.94/ 8.5 ± 1.84	30 (15/15)	7/3 8/4	FMS	Conventional rehabilitation training	45 min/s, 3 s/wk, 8wk	WCST
Zhang (29) 2025	China	-	16 (8/8)	6/2 6/2	Gamified exercise	Pretend play	30–40 min/s, 4 s/wk, 8wk	Day/night tasks, digital reverse sub-table task, dimension change card sorting task
Phung (26) 2019	USA	-	34 (14/20)	14/0 14/6	Mixed martial arts	No intervention	45 min/s, 2 s/wk, 13wk	WASI-II
Wang (20) 2025	China	7.75 ± 1.65/ 7.63 ± 1.58	22 (12/10)	-	FMS	No intervention	45 min/s, 4 s/wk, 18wk	BRIEF

T, experimental group; C, control group; W, woman; M, man; s, session; wk, week; -, not mentioned in the text; BRIEF, behavioral rating scale for executive functioning; GNG, go/no-go task; WCST, wisconsin card sorting test; BDS, backward digit span test; DSF, digit span forward; stroop test, stroop color-word test; WASI-II, wechsler abbreviated scale of intelligence - second edition; FMS, fundamental movement skills.

### 3.3 Methodological quality assessment

**Figure 2:**
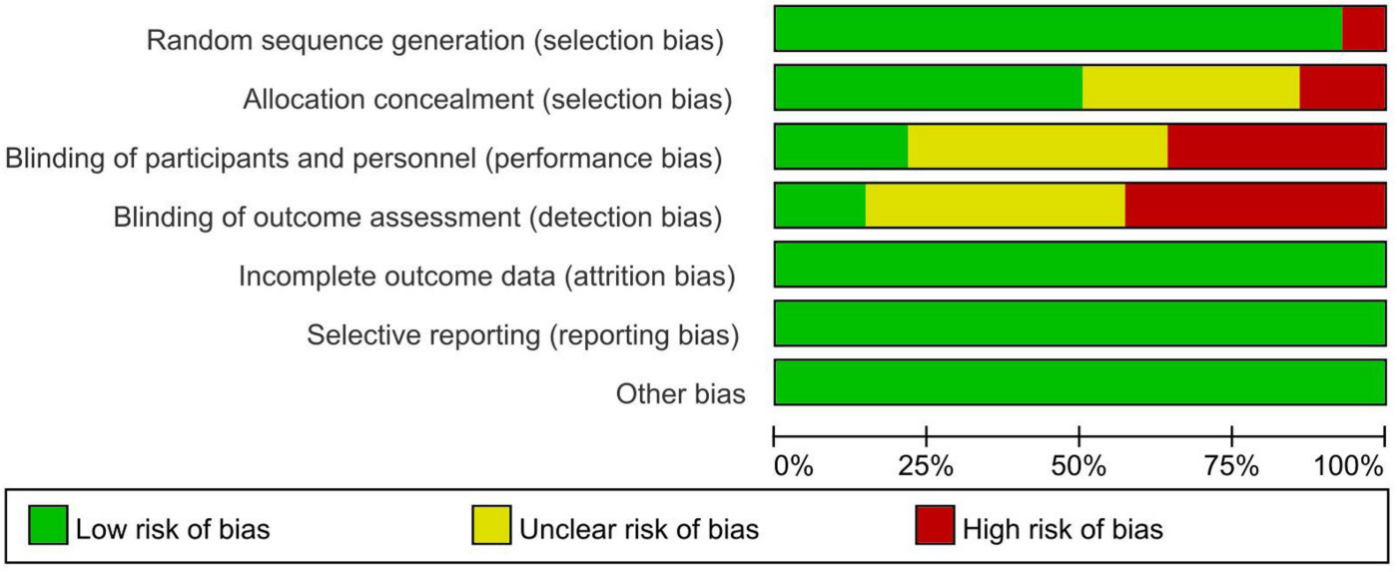
Risk of bias assessment for studies included in the analysis.

**Figure 3:**
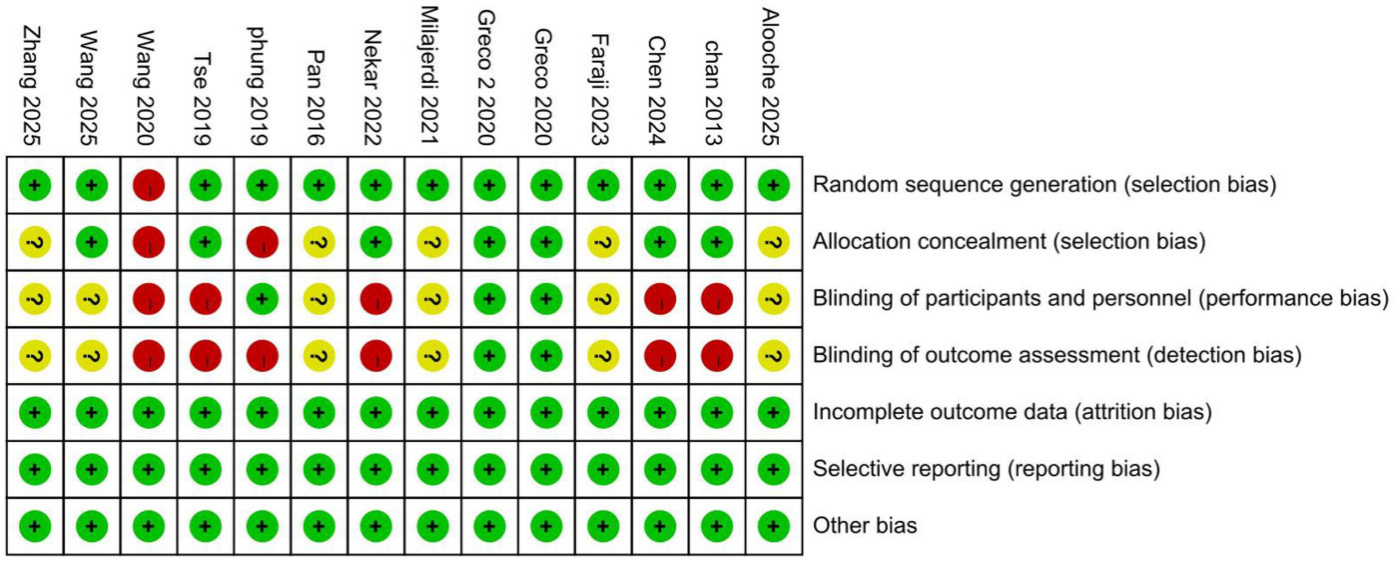
Summary of bias risk in the included studies.

The risk of bias in the included studies is shown in Figures 2, 3. Regarding selection bias, one studies (17) used block randomization, one study (18) employed random assignment by lot, one study (19) used geographical location for allocation, one study (20) used computer-generated random allocation, and the remaining studies (17, 21–26) merely stated that random allocation was used; Regarding performance bias, three studies (21, 22, 26) reported that participants were blinded, five studies (17–19, 24, 30) explicitly stated that blinding of participants was not implemented, and the remaining studies (20, 23, 25, 27–29) provided no information on this item; regarding detection bias, two studies (21, 22) detailed the use of blinding for evaluators of study results, six studies (17–19, 24, 26, 30) did not blind the outcome assessors, whereas the remaining studies (20, 23, 25, 27–29) provided no explicit information on assessor blinding; all studies provided detailed descriptions regarding attrition bias, reporting bias, and other biases.

### 3.4 GRADE

**Figure 4:**
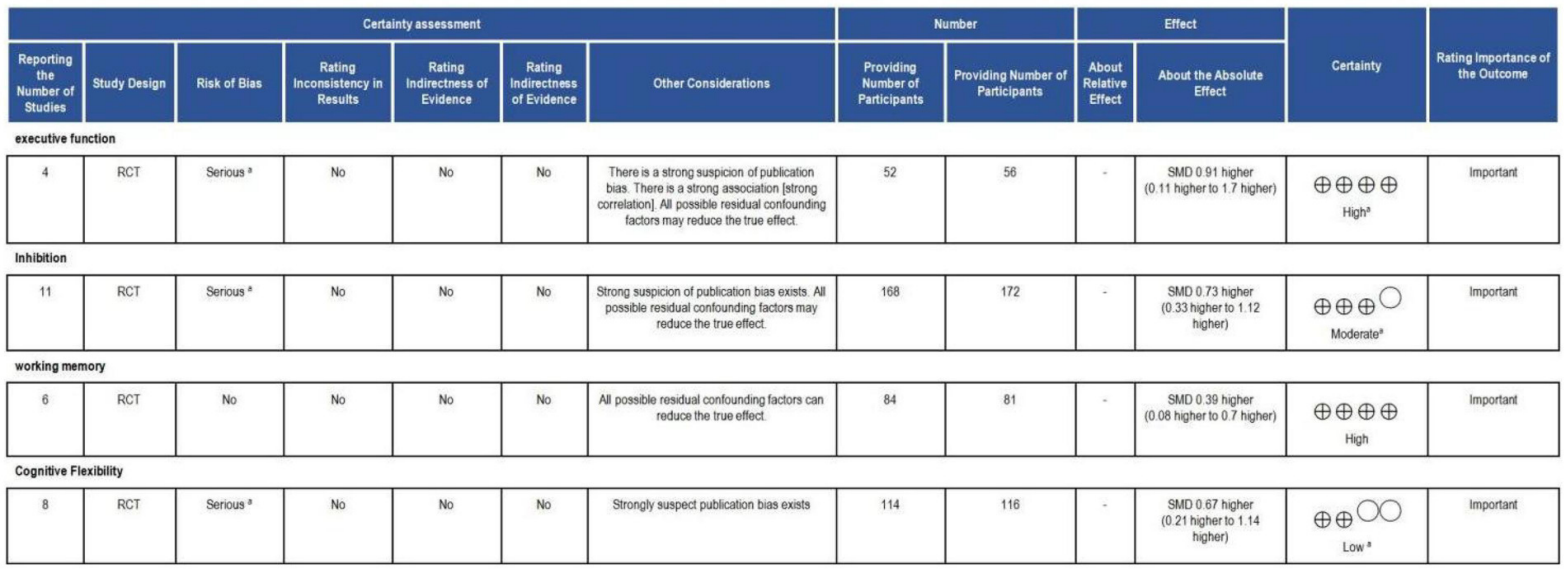
GRADE plot. CI, confidence interval; SMD, standardised mean difference; a, The study either did not blind the outcome assessors or failed to clearly report whether blinding was implemented.

Based on the GRADEpro Summary of Findings table (https://www.gradepro.org/) shown in Figure 4, we evaluated four outcomes: executive function, inhibition, working memory, and cognitive flexibility. To detail these results, executive function and working memory were assessed in four and six RCTs, respectively, with no serious inconsistencies or indirectness; therefore, evidence certainty was rated “high.” In contrast, inhibition, from 14 RCTs, was downgraded to “moderate” certainty due to lack of assessor blinding and suspected publication bias. Finally, cognitive flexibility received “low” certainty after two downgrades for inadequate blinding, imprecise effects, and high publication bias risk.

### 3.5 Meta-analysis results

**Figure 5:**
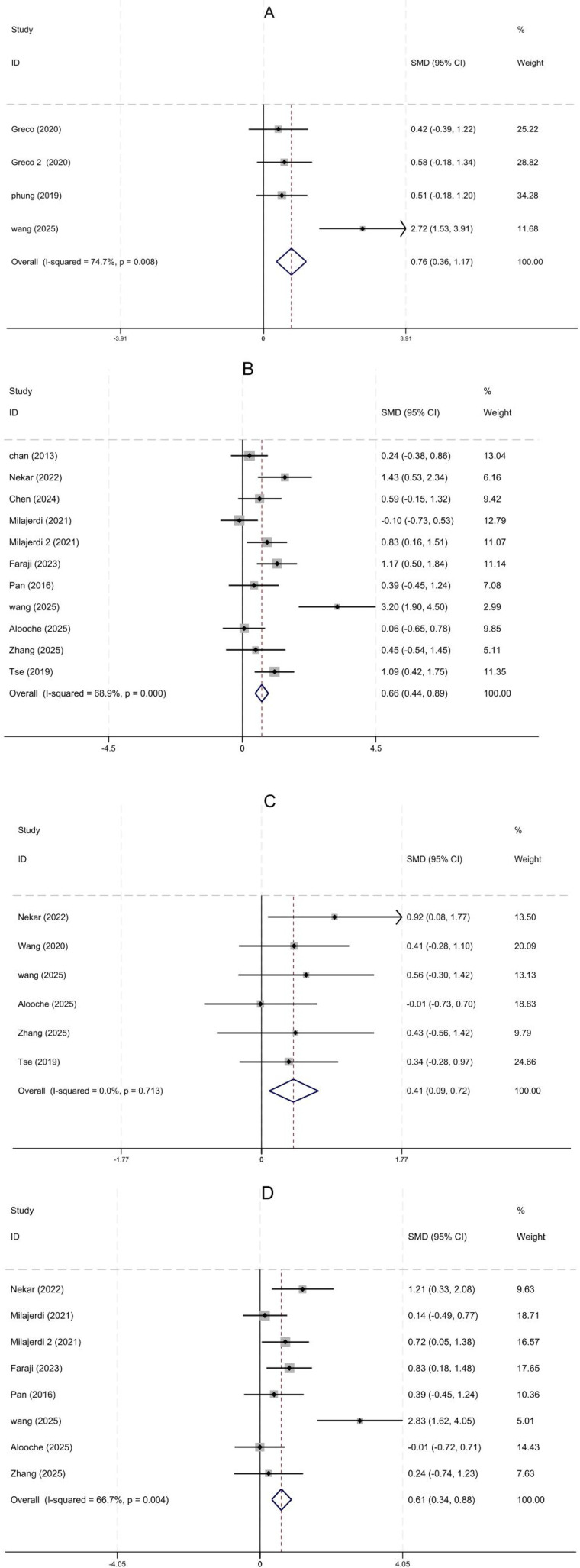
Forest plot. **(A)** Executive function; **(B)** inhibition; **(C)** working memory; **(D)** Cognitive Flexibility.

The meta-analysis results are presented in Figure 5, which incorporates 14 randomized controlled trials. Among these, four studies (20–22, 26) assessed overall EF in children and adolescents with ASD; ten studies (17, 18, 20, 23–25, 27–30) evaluated inhibitory function; six studies (17, 19, 20, 28–30) measured working memory capacity; and seven studies (20, 23, 25, 27–30) examined cognitive flexibility.

Physical activity leads to moderate-to-large improvements in overall EF, inhibition, working memory, and cognitive flexibility in children with ASD. Effects for overall EF (SMD = 0.76, 95% CI = 0.36–1.17) and inhibition (SMD = 0.66, 95% CI = 0.44–0.89, *I*^2^ = 68.9%) are comparable. Cognitive flexibility (SMD = 0.41, 95% CI = 0.09–0.72, *I*^2^ = 0%) and working memory (SMD = 0.61, 95% CI = 0.34–0.88, *I*^2^ = 66.7%) show smaller but still moderate effects. High I² values for inhibition and working memory indicate substantial heterogeneity, which may affect reliability and generalizability. We will conduct subgroup analyses to identify potential sources of this variability.

#### 3.5.1 Subgroup analysis

##### 3.5.1.1 Executive function

**Figure 6:**
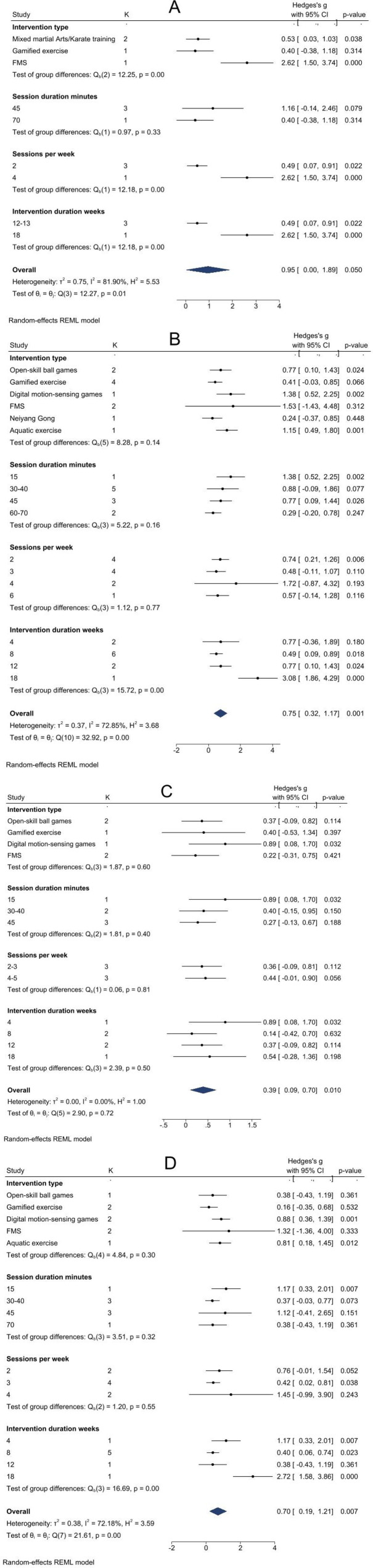
Subgroup analysis plot. **(A)** Executive function; **(B)** inhibition; **(C)** working memory; **(D)** cognitive flexibility.

Figure 6A presents heterogeneity test results for the overall EF score. Statistically significant between-group differences were observed for intervention type, weekly frequency, and duration (*p* < 0.01), indicating these factors likely contribute to heterogeneity. For exercise modality, pooled effect sizes were larger for FMS training [SMD = 2.62, 95% CI = (1.50–3.74)] and Mixed Martial Arts/Karate training [SMD = 0.53, 95% CI = (0.03–1.03)] yielded larger pooled effect sizes than controls, with FMS displaying a particularly large effect. For protocol comparison, four sessions per week for 18 weeks [SMD = 2.62, 95% CI = (1.50–3.74)] produced a greater effect size than two sessions per week for 12–13 weeks [SMD = 0.49, 95% CI = (0.07–0.91)]. Given the limited sizes of subgroups, these findings remain descriptive.

##### 3.5.1.2 Inhibition

The subgroup analysis results for inhibition are presented in Figure 6B. Specifically, the intervention period was the main source of inter-group heterogeneity (*P* = 0.00). In contrast, other grouping factors did not show statistically significant differences (*P* > 0.05), although effect sizes varied across subgroups. Regarding exercise types, and compared with the control group, significant differences in effect sizes were found for digital motion-sensing games [SMD = 1.38, 95%CI = (0.52–2.25)], aquatic exercises [SMD = 1.15, 95%CI = (0.49–1.80)], and open-skill ball games [SMD = 0.77, 95%CI = (0.10–1.43)]. Of these, digital motion-sensing games had the largest effect on improving inhibitory function in individuals with ASD.

The subgroup analysis also examined the intervention protocol. In direct comparison, a single 15-minute session [SMD = 1.38, 95%CI = (0.52–2.25)] produced a larger effect size than a single 45-minute session [SMD = 0.77, 95%CI = (0.09–1.44)]. Furthermore, regarding frequency, an intervention twice a week showed a large effect size [SMD = 0.74, 95% CI = (0.21–1.26)], whereas interventions three, four, and six times a week did not yield statistically significant differences. In terms of overall duration, 18 weeks [SMD = 3.08, 95%CI = (1.86–4.29)] yielded the largest effect size compared with 12 weeks [SMD = 0.77, 95%CI = (0.10–1.43)] and 8 weeks [SMD = 0.49, 95%CI = (0.09–0.89)], all of which were larger than the control group.

##### 3.5.1.3 Working memory

Figure 6C presents the subgroup analysis of working memory. As there was no within-group heterogeneity (*I*^2^ = 0.0%), we proceeded to compare the effects of different physical activity types and intervention protocols on working memory in children with ASD. Notably, digital motor games produced a significantly larger effect size [SMD = 0.89, 95% CI = (0.08–1.70)] than open-skill ball games, Gamified exercise, and FMS. Similarly, the protocol involving 15-minute sessions over 4 weeks also yielded the largest effect size [SMD = 0.89, 95% CI = (0.08–1.70)].

##### 3.5.1.4 Cognitive flexibility

Figure 6D shows subgroup analysis results for cognitive flexibility. Notably, intervention duration was the main source of heterogeneity within groups (*P* = 0.00). Regarding intervention types, digital motion-sensing exergames [SMD = 0.88, 95%CI = (0.36–1.39)] and aquatic exercises [SMD = 0.81, 95%CI = (0.08–1.45)] showed larger effect sizes than other types. For session frequency, both a single 15-minute session [SMD = 1.17, 95%CI = (0.33–2.01)] and three sessions per week [SMD = 0.42, 95%CI = (0.02–0.81)] produced moderate or higher effects. When considering duration, 4, 8, and 18-week interventions had greater effects than 12 weeks, with 18 weeks showing the largest effect [SMD = 2.72, 95%CI = (1.58–3.86)].

#### 3.5.2 Publication bias analysis

**Figure 7:**
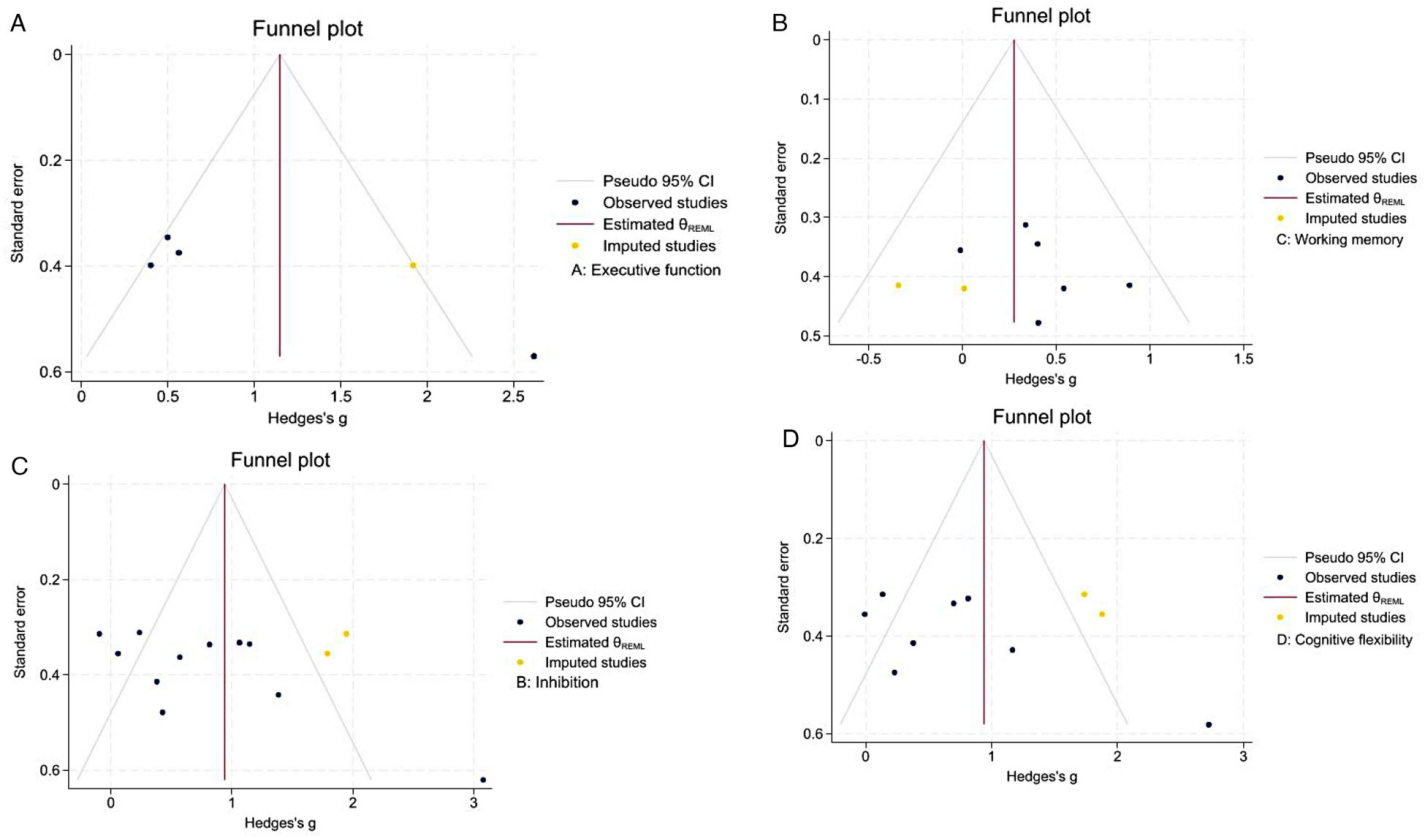
Publication bias funnel plot. **(A)** Executive function; **(B)** inhibition; **(C)** working memory; **(D)** cognitive flexibility.

To assess the reliability and robustness of the study findings, publication bias was evaluated for the included studies. For EF, Egger's regression test demonstrated statistical significance (*p* < 0.01), indicating potential publication bias or a small-sample effect. To address this, the nonparametric trim-and-fill method was applied (Figure 7A). After adding one study to the right side of the funnel plot, the plot became more symmetrical. The effect size shifted from the uncorrected pooled effect size [SMD = 0.948, 95%CI = (0.002–1.894)] to [SMD = 1.145, 95%CI = (0.309–1.981)]. These findings indicate that, despite publication bias, the positive effect of an exercise intervention on EF in children with ASD remains robust.

Egger's regression test for inhibitory ability was significant (*p* = 0.0044), indicating publication bias or small-sample effects. To address this, we used a nonparametric trim-and-fill analysis (Figure 7B). After adjustment, the effect size rose from [SMD = 0.747, 95%CI = (0.321–1.174)] to [SMD = 0.940, 95%CI = (0.498–1.383)], showing that adjusting for bias increased the estimated pooled effect size.

Egger's regression test for working memory was not significant (*p* = 0.46), and the studies showed a balanced pattern (Figure 7C), meaning a low risk of publication bias. Egger's regression test for cognitive flexibility was significant (*p* = 0.02), and the studies showed possible publication bias or small-study effects. To adjust for bias, the nonparametric trim-and-fill method was used (Figure 7D). After this, the effect size changed from [SMD = 0.70, 95%CI = (0.19–1.21)] to [SMD = 0.94, 95%CI = (0.43–1.46)]. This means that the results are fairly stable.

## 4 Discussion

A total of 14 studies were included. By carefully searching and using meta-analysis, we measured how exercise affects EF, inhibition, working memory, and cognitive flexibility in children with ASD. The findings showed that physical activity clearly improved EF and its parts in these children. However, the included studies showed significant heterogeneity in overall executive function (74.7%), inhibition (68.9%), and cognitive flexibility (66.7%). This suggests that intervention effects are influenced by specific moderating variables. Breaking the results down further, we found that the type of physical activity (e.g., fundamental movement skills, digital motion-sensing games, Aquatic exercise, or short single sessions), how often sessions occurred each week, how long each session lasted, and the total number of weeks were the main reasons for the differences. In particular, fundamental movement skills led to large improvements in EF. Programs with more frequent sessions over more weeks (4 times per week for 18 weeks) worked better than those with fewer, shorter cycles (2 times per week for 12–13 weeks). In terms of inhibition, working memory, and cognitive flexibility, digital motion-sensing games greatly improved children's performance. Aquatic exercise worked as well on inhibition as did digital motion-sensing games. Also, shorter physical activity sessions (about 15 min) worked better than longer, 45-minute sessions.

This study synthesized research on FMS training programs for children with ASD, which targeted balance, flexibility, core stability, motor coordination, and fundamental movement skills (20, 28). Notably, all interventions produced significant improvements. These results align with previous studies demonstrating that FMS is strongly correlated with EF and serves as a significant predictor of EF, inhibitory control, working memory, and cognitive flexibility (31–33). Building on this, sensorimotor integration-based training paradigms can continuously stimulate neural circuits associated with EF, including those in the prefrontal cortex, cerebellum, and basal ganglia (34–36). This stimulation, in turn, promotes neuroplasticity and enhances cognitive processes such as action planning, inhibitory control, and self-monitoring (37). Furthermore, children with ASD exhibit a significant reduction in both the total number and density of cerebellar Purkinje cells compared to typically developing peers (38). Motor coordination, motor control, and social interaction-related functions such as cognition, language, and emotion share common neural substrates within the cerebellum. As fine and gross motor skills develop, these interconnected abilities also improve (39, 40).

At the same time, regarding intervention dosage, this study found that EF in children with ASD improved more under high-frequency, long-duration intervention protocols. This observation supports the automaticity theory proposed by Shiffrin et al. The theory suggests that motor and cognitive tasks compete for a limited pool of cognitive attention resources (41). At first, motor task performance requires substantial cognitive attention. However, repeated practice leads to automatic behaviors, reducing the cognitive resources needed for execution (42). As a result, once a motor skill becomes automated, additional attention can be given to cognitive processes (43). EF is no longer engaged in the automated motor task. This change facilitates the simultaneous performance of another task with high EF demands (42).

Notably, this study found that digital motion-sensing games, such as augmented-reality cognitive-motor games and video games, produced greater improvements in inhibition, working memory, and cognitive flexibility among children with ASD. These effects were more substantial compared to those seen with ball games, combat or martial arts training, FMS, and mind-body exercises. While several studies confirm benefits across EF subdomains (30, 44), the specific effects of cognitive components vs. pure physical exercise remain debated. One potential mechanism is that autistic children may have reduced motivation and sustained attention during prolonged single-mode physical training (45). Interactive, engaging activities in digital motion-sensing games can boost motivation and attention, potentially leading to improved outcomes (46, 47). For cognitive flexibility, the diverse scene transitions and tasks in augmented reality require mental set shifting, thereby directly facilitating this skill. For working memory, overlaying virtual information onto the physical environment provides immediate memory cues, reduces cognitive workload, and makes retrieval more efficient, thereby improving memory retention (48). These clarified mechanisms explain how digital motion-sensing games enhance EF in children with autism.

This study has several limitations. The number of randomized controlled trials investigating EF interventions for children with autism remains limited, and few studies employ the same type of intervention. This scarcity may introduce bias when comparing different intervention types. Furthermore, the included studies utilized various executive function assessment tools, such as BRIEF, GNG, and WCST, which restricts direct comparability. To address differences in measurement scales, this study employed the SMD as the effect size indicator. While SMD standardizes mean differences between intervention and control groups, it presents challenges for clinical interpretation. For instance, substantial variation in baseline standard deviations across studies can obscure individual differences and potentially overestimate or underestimate the true effect, contributing to heterogeneity. These factors limit the reliability and clinical generalizability of the findings. Nevertheless, the Egger test indicated minimal publication bias, suggesting that the results are stable. Future research should increase the number and sample size of randomized trials and adopt standardized executive function assessment tools to more accurately evaluate clinical value and strengthen the reliability of research conclusions.

## 5 Conclusions

In summary, this study assessed how various physical activity and intervention protocols affect EF, inhibition, working memory, and cognitive flexibility in ASD. The results indicate that physical activity significantly improves EF and its subdomains. FMS training provided the greatest benefit for overall EF, followed by mixed martial arts or karate, with four sessions per week for 18 weeks being most effective. For inhibition, working memory, and cognitive flexibility, digital motion-sensing games that used numerical tasks had the greatest impact. A schedule of 15 min per session, two to three times per week for 18 weeks, was optimal for inhibition and cognitive flexibility, while a four-week program of 15 min per session was effective for working memory. Due to the limited number of studies, these findings should be confirmed with larger samples.

## Data Availability

The original contributions presented in the study are included in the article/Supplementary Material, further inquiries can be directed to the corresponding author.
