# Supplementary material for: Physical activity intervention improves executive function in children with autism spectrum disorder: a meta-analysis
Source: Front Pediatr. 2026 Mar 12;14:1693801. doi: 10.3389/fped.2026.1693801 (PMC13017923; doi:10.3389/fped.2026.1693801)
Supplement: Supplementary file 1 [file Table1.docx]

| Study | Assessment instrument | reverse-scored |
| --- | --- | --- |
| Tse 2019 | Go/No-Go task | The higher the error rate (or score), the poorer the inhibitory control ability. |
| Greco 2020 | BRIEF | The higher the score, the poorer the participant's executive function performance. |
| Greco2 2020 | BRIEF | The higher the score, the poorer the participant's executive function performance. |
| Wang 2020 | BRIEF | The higher the score, the poorer the participant's working memory performance. |
| Milajerdi 2021 | WCST | The higher the score, the poorer the participant's Cognitive Flexibility performance. |
| Nekar 2022 | DSF | The higher the score, the poorer the participant's working memory performance. |
| Faraji 2023 | WCST | The higher the score, the poorer the participant's Cognitive Flexibility performance. |
| Pan 2016 | WCST | The higher the score, the poorer the participant's inhibitory control and cognitive flexibility performance. |
| Zhang 2025 | day/night tasks、digital reverse sub-table task、dimension change card sorting task | The higher the scores, the poorer the participants' performance in inhibitory control, working memory, and cognitive flexibility. |
| Phung 2019 | WASI-II | The higher the score, the poorer the participant's executive function performance. |
| Wang 2025 | BRIEF | The higher the scores, the poorer the participants' performance in executive function, inhibitory control, working memory, and cognitive flexibility. |

Table Notes: BRIEF, Behavioral Rating Scale for Executive Functioning; WCST, Wisconsin Card Sorting Test; DSF, Digit Span Forward; WASI-II, Wechsler Abbreviated Scale of Intelligence - Second Edition.
