# Supplementary figures and images for: Physical activity intervention improves executive function in children with autism spectrum disorder: a meta-analysis
Source: Front Pediatr. 2026 Mar 12;14:1693801. doi: 10.3389/fped.2026.1693801 (PMC13017923; doi:10.3389/fped.2026.1693801)

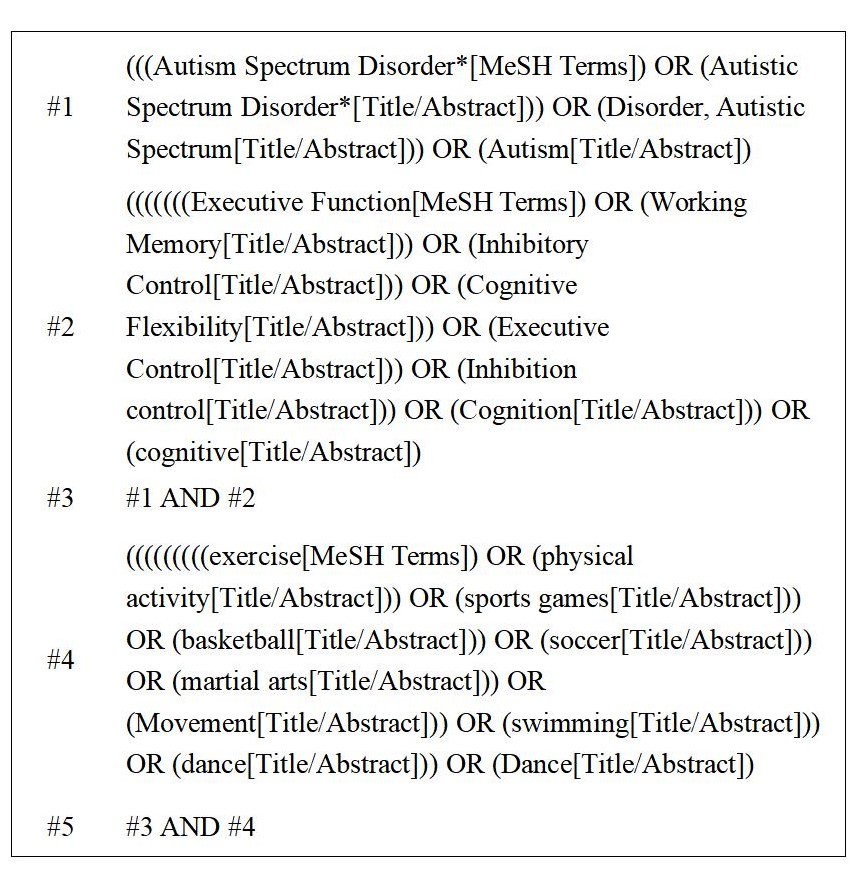

Supplement: Supplementary file 2 [file Image1.jpeg]
